# Effectiveness of Enriched Milk with Ashwagandha Extract and Tryptophan for Improving Subjective Sleep Quality in Adults with Sleep Problems: A Randomized Double-Blind Controlled Trial

**DOI:** 10.3390/clockssleep6030028

**Published:** 2024-08-09

**Authors:** Silvia Pérez-Piñero, Juan Carlos Muñoz-Carrillo, Jon Echepare-Taberna, Macarena Muñoz-Cámara, Cristina Herrera-Fernández, Vicente Ávila-Gandía, María Heres Fernández Ladreda, Javier Menéndez Martínez, Francisco Javier López-Román

**Affiliations:** 1 Faculty of Medicine, UCAM Universidad Católica San Antonio de Murcia, Carretera de Guadalupe s/n, E-9, 30107 Murcia, Spain; sperez2@ucam.edu (S.P.-P.); jcmunoz@ucam.edu (J.C.M.-C.); echepare@ucam.edu (J.E.-T.); mmunoz5@ucam.edu (M.M.-C.); cherrera@ucam.edu (C.H.-F.); vavila@ucam.edu (V.Á.-G.); 2 CAPSA, 33199 Granda, Spain; 3 Primary Care Research Group, Biomedical Research Institute of Murcia (IMIB-Arrixaca), 30120 Murcia, Spain

**Keywords:** sleep disorders, tryptophan, ashwagandha, milk, sleep quality, dietary supplementation, randomized study

## Abstract

A randomized, double-blind and controlled study was conducted to assess the effectiveness of the intake of 250 mL of lactose-free skimmed milk enriched with ashwagandha (*Withania somnifera*) alone or combined with tryptophan vs. non-enriched milk (control) on the subjective quality of sleep in healthy adults with sleep problems. The duration of supplementation was 90 days. Fifty-two eligible subjects were assigned to the study arms of ashwagandha 250 mg, ashwagandha 250 mg plus tryptophan 175 mg, ashwagandha 600 mg, and control with 13 subjects in each group. It was hypothesized that ashwagandha plus tryptophan could be superior to ashwagandha alone for improving sleep-related variables. Changes in the visual analogue scale (VAS) for sleep quality were significantly higher in the three experimental groups as compared with controls (*p* = 0.014). Improvements in the subscales of the Pittsburg Sleep Quality Index (PSQI) were found in all groups, but between-group differences were not significant. In the index of insomnia severity, decreases were higher in the three experimental groups as compared with controls especially in the group of ashwagandha 600 mg. Daytime somnolence was also reduced in the three experimental groups. Changes in anxiety levels and Morningness–Eveningness Questionnaire were not observed. The study products did not elicit changes in body composition and were well tolerated and safe. The data did not support the hypothesis, as the combination of ashwagandha and tryptophan did not show greater benefits in improving sleep quality than ashwagandha alone. However, the results from the three experimental groups containing ashwagandha were more favorable compared to the placebo group.

## 1. Introduction

Sleep is a vital physiological process that allows the body and the brain to rest and recover to perform critical functions related with neuronal development, training tasks, memory consolidation, and cardiovascular and metabolic regulatory functions. The National Sleep Foundation provided sleep duration recommendations and considered appropriate 7 to 9 h of sleep for young adults and adults, and 7 to 8 h for older adults [1]. However, there has been a decline in sleep duration and an increase in the prevalence of chronic insufficient sleep in modern societies. In a study of changes in sleep duration from 1985 to 2012 among US adults, the percentage of people sleeping ≤ 6 h increased by 31% [2]. Insufficient sleep and poor sleep quality represent a major public health concern because of the broad-ranging mental and physical well-being negative effects and safety risks [3,4,5]. Moreover, specific sleep problems are included as core symptoms across several mental disorders [6,7] and the presence of insomnia has an average prevalence of approximately 10% in multinational studies using the Diagnostic and Statistical Manual of Mental Disorders (DSM-IV) criteria [8,9].

Subjects with sleep problems are candidates for treatment with pharmacological drugs and non-pharmacological approaches. However, most commonly used insomnia medications (e.g., benzodiazepines, non-benzodiazepines, melatonin agonists, sedating antidepressants, barbiturates, orexin receptor antagonists, etc.) may develop dependency and/or adverse effects [10,11], whereas non-pharmacological options (e.g., sleep hygiene, relaxation techniques, psychotherapy, or cognitive-behavioral therapy) are generally underutilized due to scarcity of providers [12,13]. For these reasons, alternative and complementary therapies, including herbal products based on Ayurvedic medicine have gained increasing interest in the management of sleep problems.

Herbal medicine is one of the most frequently used complementary/alternative natural remedies for treating insomnia. The use of plants or plant-derived extracts for therapeutic purposes has long been used in folk medicine, with chamomile, ashwagandha (*Withania somnifera*), hops (*Humulus lupulus*), passion flower (*Passiflora incarnata* L.), lemon balm (*Melissa officinalis*), and valerian (*Valeriana officinalis*) being some of the most popular [14,15,16,17]. Biological plausibility of using these plants is based on reducing sleep latency and improving sleep duration via interactions with glutamic acid decarboxylase or via the modulation of gamma-amino butyric acid (GABA) and 5-hydroxytryptophan (5-HT) receptors [18,19].

Ashwagandha extracts from its root and leaves in the form of dietary supplements have been used for multiple purposes based on its anti-inflammatory, anti-stress, immunomodulatory, antioxidant, and rejuvenating properties [20,21] for many conditions, such as rheumatic pain, joint inflammation, cognitive disorders, anxiety and stress symptoms, male infertility, and improvement in physical performance [22,23,24,25,26]. In subjects with sleep problems, a systematic review and meta-analysis of five studies with 372 adults, treatment with ashwagandha for 6 to 12 weeks showed a significant effect on improving sleep efficiency compared with placebo, with more prominent benefits when the dose 600 mg/day was administered, when treatment duration was at least 8 weeks, and in subjects with insomnia [27]. The exact mechanism of action of ashwagandha for ameliorating sleep quality has not been fully elucidated, but besides its effects on GABAergic system [18,19], decreasing cortisol levels have been also found in different studies reported in a recent narrative review [20].

L-tryptophan is an essential amino acid that is a precursor of serotonin and melatonin, which is the main hormone involved in the control of the sleep–wake cycle. The effect of tryptophan to aid sleep has been assessed in a systematic review and meta-regression analysis showing that tryptophan supplementation, especially at ≥1 g, improved sleep quality and can shorten wake after sleep onset [28]. An increase in rated subjective sleepiness and a decrease in sleep latency associated with L-tryptophan in doses of 1 g or more have also been reported [29]. On the other hand, relatively low doses of tryptophan in the diet increased sleep performance, shortened the waking time during the night, and increased the subjective assessment of the sleep quality [30].

Based on the positive effects of both ashwagandha extracts and dietary tryptophan to improve sleep alterations previously demonstrated in previous studies [22,23,24,25,26,27,28,29], a randomized, double-blind and controlled study was designed to assess the effectiveness of enriched milk with two doses of ashwagandha (250 mg and 600 mg) and a dose of ashwagandha (250 mg) combined with tryptophan (175 mg) for improving subjective sleep quality in healthy adults with sleep problems. It was hypothesized that the sum of the combination of ashwagandha and tryptophan, both scientifically proven to improve sleep quality, could be superior to using ashwagandha alone. In this respect, the study is innovative because it was considered whether the synergy of several products can be more beneficial than consuming them individually. The intake of milk and dairy products is generally considered to promote healthy sleep [31].

## 2. Results

### 2.1. Baseline Characteristics of Participants

**Figure 1:**
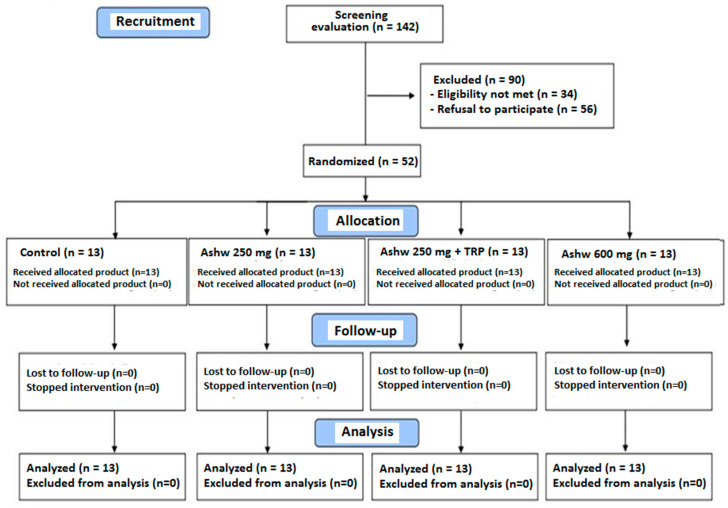
Flow chart of the study population (Ashw: ashwagandha; TRP: tryptophan).

A total of 142 subjects were initially selected to take part in the study but 90 were excluded because of ineligibility in 34 (inclusion criteria were not met) and refusal to participate in 56. The reaming 52 subjects fulfilled the inclusion criteria and completed the 90-day study period. They were randomized to the four study groups composed of 13 subjects each. The flow chart distribution of participants is shown in Figure 1.

In relation to the baseline characteristics of participants, the mean age was 26.7 ± 10.3 years and the mean weight 69.7 ± 13.6 kg. The mean VAS score of sleep quality was 3.2 ± 1.5. Differences in baseline data between the study groups were not found (Table 1).

### 2.2. Sleep Quality

#### 2.2.1. Visual Analogue Scale (VAS) Scores

Changes in VAS scores of the quality of sleep in the previous month rated by subjects assigned to the four study groups throughout the study are shown in Table 2. The sleep quality improved significantly in all study groups, but the magnitude of improvement was higher in the three experimental groups as compared with the control group. There were statistically significant differences in between-group comparisons of each experimental group versus the control group (ashwagandha 250 mg vs. control, *p* = 0.049; ashwagandha 250 mg + TRP vs. control, *p* < 0.05; ashwagandha 600 mg vs. control, *p* < 0.05) and for all four study groups (*p* = 0.014).

**Figure 2:**
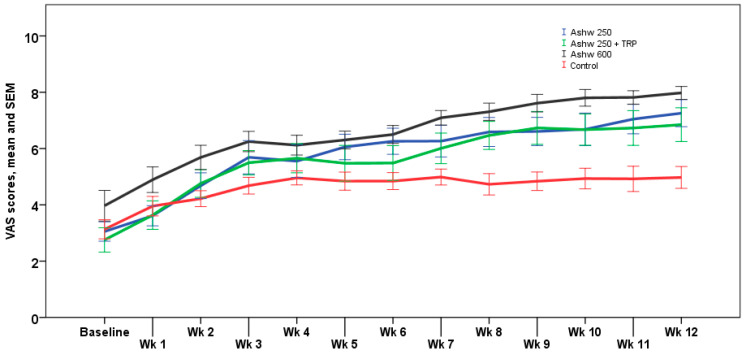
Weekly changes in VAS scores for sleep quality in the four study arms. Data expressed as mean and standard error of mean (± 1 SEM) (Wk: week).

The analysis of weekly VAS scores showed similar trends than monthly VAS scores, with significant differences at the end of the study between the three experimental groups and the control group (*p* < 0.001). The weekly trend in the control group showed a progressive increase in VAS scores from a mean of 3.1 ± 1.2 at baseline to 5.0 ± 1.4 at the end of the study, but these changes were not statistically significant. In the ashwagandha 250 mg group, the increase in VAS scores was statistically significant from the 4th week of treatment until the end of the study (*p* < 0.001) with an absolute mean increase of 4.3 cm. In the ashwagandha 250 mg + TRP group, increases in VAS scores achieved significant differences from the first week of treatment, which remained until the end of the study (*p* < 0.001), with an absolute mean increase of 4.0 cm. Finally, in the ashwagandha 600 mg group, VAS increases were statistically significant from the 2nd week until the end of the study (*p* < 0.001) with an absolute mean increase of 4.1 cm. Weekly VAS values registered in the diary card in the four study arms are shown in Figure 2.

#### 2.2.2. Pittsburg Sleep Quality Index (PSQI) Scores

Results obtained in the different subscales of the PSQI questionnaire in the four study groups are shown in Table 3. In all study groups, there was a statistically significant decrease in mean values at the end of the study as compared with baseline for all subscales of the questionnaire except for sleep efficiency. However, the comparisons of between-group differences for the decreases found in all study groups were not statistically significant in any subscale.

### 2.3. Severity of Insomnia

**Figure 3:**
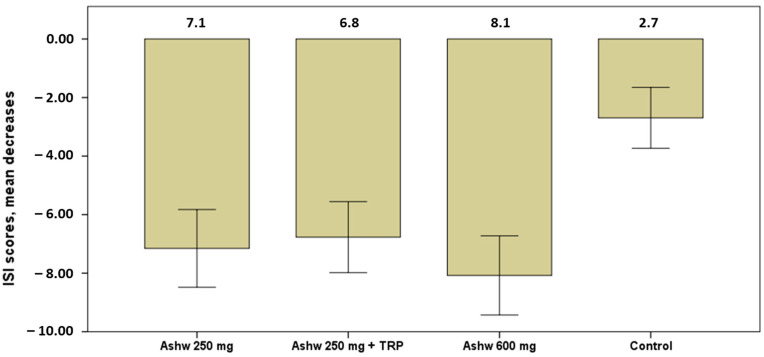
Decrease in mean scores of the severity of insomnia in the four study groups, with higher significant decreases in the three experimental groups especially in the ashwagandha 600 mg group (mean 8.1) vs. the control group (*p* < 0.001). Data expressed as mean and standard error of mean (± 1 SEM) (Ashw: ashwagandha; TRP: tryptophan).

The severity of insomnia showed significant decreases in the mean scores of the ISI questionnaire in all groups at the end of the study as compared with baseline (Table 4). Decreases, however, were higher in the three experimental groups as compared with the control group, particularly among subjects assigned to the group of ashwagandha 600 mg (Figure 3).

### 2.4. Epworth Sleepiness Scale

The mean scores of the Epworth sleepiness scale decreased in all study groups at the end of the study as compared baseline, but the differences were statistically significant in the three experimental groups only (Table 5). Between-group differences were statistically significant (*p* = 0.050), but reduction in daytime somnolence was significantly higher among subjects assigned to the ashwagandha 250 mg than in those assigned to the control group (*p* < 0.05).

### 2.5. Morningness–Eveningness Questionnaire (MEQ)

In relation to chronotypes, all subjects were considered “intermediate types” according to the mean scores of the MEQ questionnaire. As shown in Table 6, changes were not statistically significant, except for a small increase in the mean scores of the MEQ in the ashwagandha 600 mg group.

### 2.6. Anxiety

In relation to anxiety levels, subjects assigned to the experimental groups showed a statistically significant decrease in scores in the STAI-state scale at the end of the study as compared with baseline, whereas changes in the control group were not significant (Table 7). However, between-group comparison did not reveal significant differences (*p* = 0.378). In the STAI-trait subscale, changes at the end of the study as compared with baseline were statistically significant in the four study groups, but between-group differences did not reach statistical significance (*p* = 0.946) (Table 7).

### 2.7. Anthropometric Variables

The body composition assessed by BIA did not vary during the 90 days of the study in any of the study groups (Table 8). None of the within-group and between-group comparisons for weight, BMI, percentage of fat mass, and muscle mass were statistically significant.

### 2.8. Compliance and Safety

All participants consumed at least 80% of the study product. The maximum number of milk cartons returned was 4, with compliance ranging between 100% and 83.3%. Changes in systolic and diastolic blood pressure and heart rate during the study period were not observed. Also, the results of laboratory analyses (complete blood cell count, liver function tests, and renal function tests) remained within the normal ranges, which confirmed the safety of the investigational products. AEs of mild-moderate intensity were reported by three participants in the experimental groups (7.7%). Two subjects assigned to the ashwagandha 600 mg group reported symptoms of poor digestion and abdominal bloating, and one subject treated with ashwagandha 250 mg and tryptophan reported poor digestion.

## 3. Discussion

This study aimed to assess if the effect of milk enriched with different active products including ashwagandha 250 mg, ashwagandha 250 and tryptophan, or ashwagandha 600 mg, consumed for 90 consecutive days was associated with a significant improvement in the subjective perception of better sleep quality as compared with participants assigned to non-enriched milk (control). The data did not support the hypothesis, as the combination of ashwagandha and tryptophan did not show greater benefits in improving sleep quality than ashwagandha alone. However, the results from the three experimental groups containing ashwagandha were more favorable compared to the placebo group. Differences in VAS scores in favor of the experimental groups were noted in perceptions of sleep quality at the mid-study and final visits, which were consistent with weekly assessments of improvement as recorded in the sleep diary cards. Increases in daily VAS scores were already recorded from the 1st and 2nd weeks of treatment among subjects assigned to the experimental groups.

Interestingly, subjective improvement in the quality of sleep was not evident when different sleep-related parameters were quantified using standard questionnaires. This may suggest that there is a difference between measurements of sleep efficiency using objective tools and people’s own perception of their sleep quality and in how they link good sleep to their well-being. In a study that investigated how self-reported and actigraphy-measured sleep parameters influenced subjective well-being and life satisfaction in 109 university students, when participants reported that they slept better than they normally did, they experienced more positive emotions and had a higher sense of life satisfaction the following day, but this feeling was unrelated to actigraphy-derived measure of sleep efficiency [32]. Another study in 147 individuals aged 18–25 years old who received seven prompts per day, answered questions about their affective states and reported their sleep every morning, better sleep quality was associated with the next morning’s affective well-being [33].

Other findings of the study include the improvement in the severity of insomnia in subjects assigned to milks enriched with ashwagandha 250 mg and 600 mg, particularly at doses of 600 mg, as well as a reduction in daytime sleepiness. These results are consistent with research studies showing that ashwagandha supplements can act as a sleep aid in subjects with insomnia symptoms [20,27,34,35,36,37]. Studies in animal models have shown that ashwagandha root extracts exert sleep-promoting activity through the GABAergic system [38,39].

The essential amino acid L-tryptophan is often used to treat insomnia but data from clinical studies of the combination of ashwagandha and tryptophan for improving sleep quality in subjects with sleep disturbances have not been previously reported. In our study, the results obtained in the experimental arm of ashwagandha 250 mg and tryptophan 175 mg were not superior to those obtained in the experimental arms with ashwagandha 250 and 600 mg. On the other hand, none of the active products included in the experimental groups showed an effect on the levels of anxiety or chronotypes of participants, although supplementation with ashwagandha extracts has been reported to reduce perceived stress and anxiety symptoms [8,40].

The present findings should be interpreted considering the limitations of the study especially the reduced number of participants in each study arm, which affects the generalizability of results, and the duration of the dietary intervention of 90 days. According to data reported by the National Institutes of Health regarding the implications for use of ashwagandha, the product appears to be well tolerated for up to 3 months of use [41]. Also, the involvement of GABA receptors in the pharmacological mechanisms of action of ashwagandha extract and Z-drugs might influence sharing long-term effects of tolerance and dependence. This potential long-term effect of ashwagandha extract could not be evaluated in the present study as the use of the investigational product was limited to 90 days. The efficacy and safety of long-term ashwagandha use over months or years for stress, anxiety, or sleep is not known [41].

Other methods to collect objective data of sleep quality, such as polysomnography or actigraphy were not used. However, strengths of the study include the randomized, double-blind, and controlled design and the use of ashwagandha at two different doses and in combination with tryptophan. In addition, the study products did not elicit changes in body composition and were well tolerated and safe. The use of ashwagandha combined with tryptophan did not appear to be superior to ashwagandha alone, particularly in comparison with the high dose of 600 mg. However, the study did not allow for a head-to-head comparison of tryptophan and ashwagandha extract, which would be of interest for future clinical research. Finally, it is unknown whether the melissa extract contained in the skimmed milk of the three experimental products could have influenced the subjective sleep quality measured by VAS.

## 4. Materials and Methods

### 4.1. Study Design and Participants

A single-center, randomized, double-blind, four-arm parallel-group, and controlled study was designed and conducted at the Health Sciences Department of Universidad Católica San Antonio de Murcia (UCAM), in Murcia, Spain. The study began in 14 March 2023 and finished in 20 June 2023. The primary objective of the study was to assess the effectiveness of dietary supplementation with different types of enriched milk consumed during 90 days on the quality of sleep in subjects with sleep disturbances. Secondary objectives included changes in sleep quality evaluated using the Pittsburgh Sleep Quality Index (PSQI), anxiety levels, subjective feeling of insomnia, level of daytime sleepiness, and the sleep–wake cycle, as well as to evaluate tolerability and safety of the study products.

Participants were mainly recruited by advertising the study through mass media and social networks available at UCAM University. Inclusion criteria were healthy adults aged 18 years or older, who complained of poor sleep quality defined as score > 5 in the PSQI questionnaire, and able to understand the clinical study and willing to comply with the study procedures and requirements. Exclusion criteria were presence of severe or terminal illness; body mass index (BMI) > 32 kg/m^2^; use of medications that may affect cognitive function or sleep, such as anticonvulsants, barbiturates, benzodiazepines, antidepressants, neuroleptics, alcohol, and illicit drugs; organic dementias, such as Alzheimer’s disease, Huntington’s disease, Parkinson’s disease, and senile dementia; known allergy or hypersensitivity to any of the study components; pregnant or breast-feeding women; participation in another clinical study involving blood sampling or dietary interventions; inability to understand the informed consent.

The study protocol was approved by the Ethics Committee of Universidad Católica San Antonio de Murcia (code CE022312 approval date 24 February 2023) (Murcia, Spain) and was registered in the ClinicalTrials.gov (NCT06154616). Written informed consent was obtained from all participants.

### 4.2. Randomization and Intervention

Participants were randomly assigned in equal proportion (1:1:1:1) to one of the four study arms by a blind investigator using a simple randomization procedure with the Epidat 4.1 software program. The study arms included three experimental groups (a, b, c) and one control group (d) and were as follows: (a) lactose-free skimmed milk enriched with 250 mg ashwagandha, (b) lactose-free skimmed milk enriched with 250 mg ashwagandha and 175 mg tryptophan (TRP), (c) lactose-free skimmed milk enriched with 600 mg ashwagandha, and d) lactose-free skimmed milk (control). The skimmed milk of the three experimental groups (a, b, c) contained 300 mg of melissa (*Melissa officinalis*) extract, 1.4 mg of vitamin B6, and 200 µg of vitamin B9. Subjects assigned to the control group received lactose-free skimmed milk only.

The study products had the same organoleptic characteristics and were delivered to participants in single identical milk cartons of 250 mL. Participants were instructed to take the study product 30 min before bedtime for 90 consecutive days. Compliance with the study product in percentage was defined as the number of milk cartons taken by participants during the study divided by the number of cartons expected to have been taken (*n* = 90) multiplied by 100. Participants were required to consume at least 80% of milk cartons, so that only 4.5 milk cartons could be left corresponding to 18 days out of 90 days of consumption. It was strongly recommended not to make changes to the dietary habits. The use of any new medication should be reported to the principal investigator, and any other medications taken during the course of the study should be recorded in the diary card.

### 4.3. Study Procedures

Participants were visited at the study center within ± 10 days of the baseline visit, in which eligible subjects were selected and the written informed consent was obtained. The study period included three visits, a baseline visit (visit 1, day 0), and intermediate visit (visit 2, days 45 ± 2), and a final visit (visit 3, day 90).

At the baseline visit (visit 1), eligibility criteria were confirmed, a medical history was taken, and subjects were randomized to the study groups. Other procedures included assessment of sleep quality using a visual analogue scale (VAS); administration of the PSQI questionnaire, the Epworth sleepiness scale, the Morningness–Eveningness Questionnaire, the State-Trait Anxiety Inventory (STAI) questionnaire, and the Insomnia Severity Index (ISI); assessment of body composition by bioelectrical impedance analysis (BIA). The study product for the first half of the study period was provided together with a diary card in which subjects had to register their perception of sleep quality at night.

At the mid-study visit (visit 2), sleep quality using VAS was evaluated, the assigned study product for the next 45 days of treatment was provided, and adverse events (AEs) were recorded. Also, the remaining study product was collected and compliance was calculated. At the final visit at 90 days (visit 3), the same procedures as those of the baseline visit were performed. Compliance with the study product and AEs were recorded.

### 4.4. Study Variables

Sleep quality referred to the past month was evaluated at each study visit using a 10-cm unnumbered VAS scale, where 0 was “very poor sleep quality” and 10 “very good sleep quality”. The score was determined by measuring the distance on the 10-cm line between the ‘very poor sleep quality’ anchor and the subject’s mark. VAS scores < 4 indicated a poor sleep quality, scores between 4 and 6 a moderate–good sleep quality, and scores > 6 a good sleep quality. Subjects were also instructed to assess their quality of sleep at night, every morning when getting out of bed using a VAS score and to register daily data in the diary card.

The PSQI is a self-rated 19-item questionnaire that assesses sleep quality over an interval of 1 month. These items are combined into seven components (subjective sleep quality, sleep latency, duration of sleep, sleep efficiency, sleep disturbances [awakenings], daytime dysfunction, and need of sleep medication), which are scored from 0 (no difficulty) to 3 (severe difficulty). The domain of need of sleep medication was not evaluated in this study. The overall score ranges between 0 and 21, with higher scores indicating worse sleep quality. A Spanish validated version of the PSQI was used [42].

The Insomnia Severity Index (ISI) is a 7-item self-report questionnaire assessing the nature, severity, and impact of insomnia. The usual recall period is the “last month” and the dimensions evaluated are: severity of sleep onset, sleep maintenance, and early morning awakening problems, sleep dissatisfaction, interference of sleep difficulties with daytime functioning, noticeability of sleep problems by others, and distress caused by the sleep difficulties. A 5-point Likert scale is used to rate each item (0 = no problem; 4 = very severe problem), yielding a total score ranging from 0 to 28, with 0–7 interpreted as absence of insomnia, 8–14 sub-threshold insomnia, 15–21 moderate insomnia, and 22–28 severe clinical insomnia. A Spanish validated version of ISI was administered [43].

The Epworth sleepiness scale includes a list of seven situations, in which the subject refers the tendency to become sleepy on a scale of 0, no chance of dozing to 3, high chance of dozing. The total score is based on a scale of 0 to 24. Results 0 to 10 indicate normal daytime sleepiness, 11 to 15 mild-moderate excessive daytime sleepiness, and 16 to 24 severe excessive daytime sleepiness. A Spanish validated version was used [44].

The Morningness–Eveningness Questionnaire (MEQ) is a 19-item self-reported assessment of morningness and eveningness preferences. Questions are multiple-choice, framed in a preferential manner, and rated as 4–5 point numerical scale. The sum gives a score ranging from 16 to 86; scores of 41 and below indicate “evening types”, scores of 59 and above indicate “morning types”, scores between 42 and 58 indicate “intermediate types”. A Spanish validated version of the MEQ was used [45].

The STAI questionnaire measures state (STAI-state) and trait (STAI-trait) of anxiety based on 20 questions for each domain, scores can vary between 0 and 60 with higher scores indicating greater anxiety levels. Scores 0–9 indicate normal or no anxiety, 10–18, mild to moderate anxiety, 19–29, moderate to severe anxiety, and 30–60, severe anxiety. A Spanish validated version was used [46].

A whole body bioimpedance analyzer (Tanita BC-420MA, Tanita Corp., Tokyo, Japan) was used to determine corporal composition (weight, BMI, percentage of fat mass, and muscle mass expressed in kg). The use of concomitant medication was also registered. Safety data included blood pressure recording and laboratory analyses, which were performed at baseline and at the end of the study. Standard hematological (hemogram) and biochemical parameters (renal and liver function tests) were measured.

### 4.5. Study Endpoints

The primary endpoint of the study was the change from baseline to the end of the study in the subjective quality of sleep measured by VAS in the experimental arms as compared with the control arm as well as between the experimental groups among each other. Secondary endpoints were changes in the study instruments (PSQI, ISI, Epworth sleepiness scale, MEQ, and STAI) after 90 days of consumption of enriched milk. Safety endpoints were anthropometric variables, vital signs, laboratory values, and AEs.

### 4.6. Statistical Analysis

The sample size was calculated based on the mean change in the quality of sleep measured by VAS as the primary endpoint of the study. Considering a standard deviation (SD) of 1.59 of the mean post-treatment VAS score reported in a similar population [47], for a precision of 1.6 with an alpha risk of 5% and a statistical power of 80%, 11 subjects in each group were needed. If we consider a 10% loss to follow-up, 13 subjects in each group would be required.

Data of all participants who met the eligible criteria and completed the 90-day study period were analyzed. Categorical data are expressed as frequencies and percentages, and continuous data as mean and standard deviation (± SD). Differences in the distribution of variables between the experimental and control groups were analyzed with the chi-square test for qualitative variables and the Student’s *t* test for quantitative variables. The analysis of variance (ANOVA) for repeated measures was used to assess the change in variables corresponding to each group throughout the study period. The subject factor included data at baseline, at the mid-study visit (45 days), and at the final visit (90 days) and the between-subject factor for paired data included the product administered, namely, milk enriched with 250 mg ashwagandha, milk enriched with 250 mg ashwagandha and TRP, milk enriched with 600 mg ashwagandha, or non-enriched control milk. Turkey’s or Bonferroni’s correction was applied for post-hoc analyses. To calculate intergroup differences, the analysis of covariance (ANCOVA) was used, with the product consumed as the intersubject factor and the initial value of the variable as covariate. A *p* < 0.05 was considered statistically significant. Data were analyzed with the SPSS version 25.0 (IBM Corp., Armonk, NY, USA) software program.

## 5. Conclusions

In healthy subjects with sleep problems, the administration of a dietary supplement based on milk enriched with ashwagandha alone or combined with tryptophan during 90 days improved significantly the subjective perception of sleep quality and the severity of insomnia. Both doses of ashwagandha of 250 mg and 600 mg were effective, although the magnitude of the effect seems to be higher with the 600 mg dose. Further studies with larger sample sizes would be helpful to validate the results and enhance the robustness of findings. The individual contribution of tryptophan merits also to be evaluated in future studies.

## Figures and Tables

**Table 1:** Baseline characteristics of the study population.

Variables	Control (*n* = 13)	Ashw 250 mg (*n* = 13)	Ashw 250 mg + TRP (*n* = 13)	Ashw 600 mg (*n* = 13)	Total (*n* = 52)
Gender, men/women	4/9	2/11	6/7	4/9	16/36
Age, years	26.5 ± 10.3	26.3 ± 10	26.9 ± 10.4	25.3 ± 9.3	26.7 ± 10.3
Weight, kg	64.2 ± 13.6	69.8 ± 13.9	74.5 ± 10.9	70.3 ± 15.3	69.7 ± 13.6
BMI, kg/m^2^	23.2 ± 3.2	25.2 ± 4.1	24.5 ± 2.3	24.5 ± 3.8	24.4 ± 3.4
VAS score sleep quality	3.1 ± 1.2	3.0 ± 1.2	2.8 ± 1.6	4.0 ± 2.0	3.2 ± 1.5

Ashw: ashwagandha; TRP: tryptophan; BMI: body mass index; VAS: visual analogue scale. Data as mean ± standard deviation.

**Table 2:** Changes in visual analogue scale (VAS) scores of sleep quality in the four study groups.

Study Groups	Visit 1 Baseline	Visit 2 Mid-Study (45 days)	Visit 3 Final (90 days)	Within-Group Differences Visits 1 vs. 3 *p* Value	Between-Group Differences *p* Value (η^2^)
Control	3.1 ± 1.2	4.4 ± 1.2	5.0 ± 1.4	0.006	0.014 (0.15)
Ashw 250 mg	3.0 ± 1.2	6.2 ± 1.6	7.2 ± 1.6 #	<0.001	
Ashw 250 mg + TRP	2.8 ± 1.6	5.8 ± 2.0	6.7 ± 2.1 #	<0.001	
Ashw 600 mg	4.0 ± 2.0	6.6 ± 1.3	7.9 ± 0.9 ##	<0.001	

Ashw: ashwagandha; TRP: tryptophan. Data as mean ± standard deviation. η^2^: effect size. # *p* < 0.05; ## *p* < 0.01, significant intergroup differences with respect to the control group (ANCOVA).

**Table 3:** Changes in the different subscales of the PSQI questionnaire during the study period in the four treatment arms.

Study Groups	Visit 1 Baseline	Visit 3 Final (90 days)	Within-Group Differences Visits 1 vs. 3 *p* Value	Between-Group Differences *p* Value (η^2^)
Overall score				
Control	9.9 ± 2.0	6.9 ± 2.3	0.001	0.241 (0.083)
Ashw 250 mg	10.8 ± 3.4	5.3 ± 2.3	0.001	
Ashw 250 mg + TRP	10.1 ± 2.7	5.5 ± 1.9	0.001	
Ashw 600 mg	10.1 ± 2.4	5.5 ± 1.8	0.001	
Sleep quality				
Control	2.0 ± 0.4	1.4 ± 0.8	0.007	0.226 (0.086)
Ashw 250 mg	2.2 ± 0.4	1.0 ± 0.7	0.001	
Ashw 250 mg + TRP	2.2 ± 0.6	1.0 ± 0.7	0.001	
Ashw 600 mg	2.1 ± 1.0	0.9 ± 0.5	0.001	
Sleep latency				
Control	2.2 ± 0.8	1.7 ± 1.0	<0.05	0.382 (0.061)
Ashw 250 mg	2.2 ± 0.8	1.4 ± 0.8	0.005	
Ashw 250 mg + TRP	2.3 ± 0.8	1.5 ± 0.9	0.009	
Ashw 600 mg	2.5 ± 1.8	1.3 ± 0.9	0.001	
Sleep duration				
Control	1.5 ± 0.8	0.9 ± 0.8	0.013	0.450 (0.053)
Ashw 250 mg	2.2 ± 1.0	1.2 ± 0.7	0.001	
Ashw 250 mg + TRP	1.8 ± 0.7	0.9 ± 0.7	0.009	
Ashw 600 mg	2.1 ± 1.2	0.9 ± 0.6	0.001	
Sleep efficiency, %				
Control	78.4 ± 12.5	81.5 ± 10.3	0.493	0.939 (0.008)
Ashw 250 mg	72.8 ± 18.2	78.8 ± 8.1	0.194	
Ashw 250 mg + TRP	79.6 ± 14.2	84.7 ± 5.7	0.266	
Ashw 600 mg	74.6 ± 15.2	81.7 ± 10.5	0.126	
Disturbances, number				
Control	12.2 ± 4.3	9.2 ± 4.0	0.019	0.301 (0.073)
Ashw 250 mg	13.4 ± 5.2	7.1 ± 2.9	0.001	
Ashw 250 mg + TRP	12.0 ± 6.4	7.0 ± 3.3	0.001	
Ashw 600 mg	12.2 ± 2.3	6.9 ± 2.4	0.001	
Daytime dysfunction				
Control	1.8 ± 0.6	1.2 ± 0.7	0.007	0.558 (0.042)
Ashw 250 mg	1.9 ± 1.0	1.0 ± 0.6	0.001	
Ashw 250 mg + TRP	2.0 ± 1.0	1.0 ± 0.7	0.001	
Ashw 600 mg	2.1 ± 0.8	1.1 ± 0.6	0.001	

Ashw: ashwagandha; TRP: tryptophan. Data expressed as mean ± standard deviation. η^2^: effect size.

**Table 4:** Changes in the scores of the ISI questionnaire in the study groups during the study.

Study Groups	Visit 1 Baseline	Visit 3 Final (90 days)	Within-Group Differences Visits 1 vs. 3 *p* Value	Between-Group Differences *p* Value (η^2^)
Control	17.1 ± 4.2	14.4 ± 4.3	0.035	0.018 (0.188)
Ashw 250 mg	17.3 ± 4.6	10.2 ± 3.8	0.001	
Ashw 250 mg + TRP	17.8 ± 3.7	11.0 ± 3.8	0.001	
Ashw 600 mg	17.1 ± 3.1	9.0 ± 4.0	0.001	

Ashw: ashwagandha; TRP: tryptophan. Data expressed as mean ± standard deviation. η^2^: effect size.

**Table 5:** Daytime somnolence measured with the Epworth sleepiness scale in the four study groups.

Study Groups	Visit 1 Baseline	Visit 3 Final (90 days)	Within-Group Differences Visits 1 vs. 3 *p* Value	Between-Group Differences *p* Value (η^2^)
Control	8.5 ± 4.1	7.9 ± 3.8	0.550	0.050 (0.131)
Ashw 250 mg	13.4 ± 5.6	8.5 ± 3.0 #	0.001	
Ashw 250 mg + TRP	11.5 ± 5.2	7.7 ± 3.5	0.002	
Ashw 600 mg	11.9 ± 4.8	8.9 ± 5.1	0.010	

Ashw: ashwagandha; TRP: tryptophan. Data expressed as mean ± standard deviation. η^2^: effect size # *p* < 0.05, significant intergroup differences with respect to the control group (ANCOVA).

**Table 6:** Chronotypes of the study population as shown by the MEQ scale.

Study Groups	Visit 1 Baseline	Visit 3 Final (90 days)	Within-Group Differences Visits 1 vs. 3 *p* Value	Between-Group Differences *p* Value (η^2^)
Control	42.1 ± 23.2	45.1 ± 11.8	0.142	0.907 (0.011)
Ashw 250 mg	48.1 ± 11.8	50.5 ± 10.7	0.226	
Ashw 250 mg + TRP	44.5 ± 11.5	46.5 ± 10.1	0.324	
Ashw 600 mg	46.7 ± 9.3	50.7 ± 11.2	0.050	

Ashw: ashwagandha; TRP: tryptophan. Data expressed as mean ± standard deviation. η^2^: effect size.

**Table 7:** Changes in anxiety levels in the four study groups during the study period.

Study Groups	Visit 1 Baseline	Visit 3 Final (90 days)	Within-Group Differences Visits 1 vs. 3 *p* Value	Between-Group Differences *p* Value (η^2^)
STAI-state				
Control	19.1 ± 7.6	14.5 ± 6.5	0.100	0.378 (0.062)
Ashw 250 mg	30.9 ± 11.3	19.9 ± 7.2	0.001	
Ashw 250 mg + TRP	23.6 ± 15.0	14.9 ± 13.1	0.002	
Ashw 600 mg	28.4 ± 12.4	18.9 ± 8.9	0.001	
STAI-trait				
Control	25.9 ± 8.9	19.9 ± 7.8	0.001	0.946 (0.008)
Ashw 250 mg	31.8 ± 11.4	24.9 ± 9.7	0.001	
Ashw 250 mg + TRP	27.2 ± 13.5	19.9 ± 11.1	0.001	
Ashw 600 mg	28.6 ± 8.4	22.5 ± 8.5	0.002	

Ashw: ashwagandha; TRP: tryptophan. Data expressed as mean ± standard deviation. η^2^: effect size.

**Table 8:** Changes in body composition during the study period in the four study groups.

Study Groups	Visit 1 Baseline	Visit 3 Final (90 days)	Within-Group Differences Visits 1 vs. 3 *p* Value	Between-Group Differences *p* Value
Weight, kg				
Control	64.2 ± 13.6	64.5 ± 13.3	0.521	0.762
Ashw 250 mg	69.8 ± 13.9	69.5 ± 13.9	0.479	
Ashw 250 mg + TRP	74.5 ± 10.9	74.7 ± 11.9	0.705	
Ashw 600 mg	70.3 ± 15.3	70.5 ± 16.2	0.610	
BMI, kg/m^2^				
Control	23.2 ± 3.2	23.3 ± 3.3	0.439	0.718
Ashw 250 mg	25.2 ± 4.1	25.1 ± 4.2	0.439	
Ashw 250 mg + TRP	24.5 ± 2.4	24.5 ± 2.7	0.923	
Ashw 600 mg	24.5 ± 3.8	24.6 ± 4.0	0.662	
Fat mass, %				
Control	22.9 ± 8.5	22.5 ± 8.1	0.237	0.816
Ashw 250 mg	29.7 ± 7.0	29.6 ± 6.6	0.872	
Ashw 250 mg + TRP	25.9 ± 8.2	25.6 ± 8.2	0.399	
Ashw 600 mg	25.8 ± 6.9	25.8 ± 7.2	0.984	
Muscle mass, kg				
Control	46.8 ± 10.6	47.3 ± 10.6	0.159	0.671
Ashw 250 mg	46.0 ± 6.8	45.9 ± 7.4	0.813	
Ashw 250 mg + TRP	52.7 ± 11.0	53.0 ± 11.4	0.345	
Ashw 600 mg	49.3 ± 11.3	49.5 ± 11.7	0.698	

Ashw: ashwagandha; BMI: body mass index; TRP: tryptophan. Data expressed as mean ± standard deviation.

## Data Availability

Research data are available from the corresponding author upon request.
